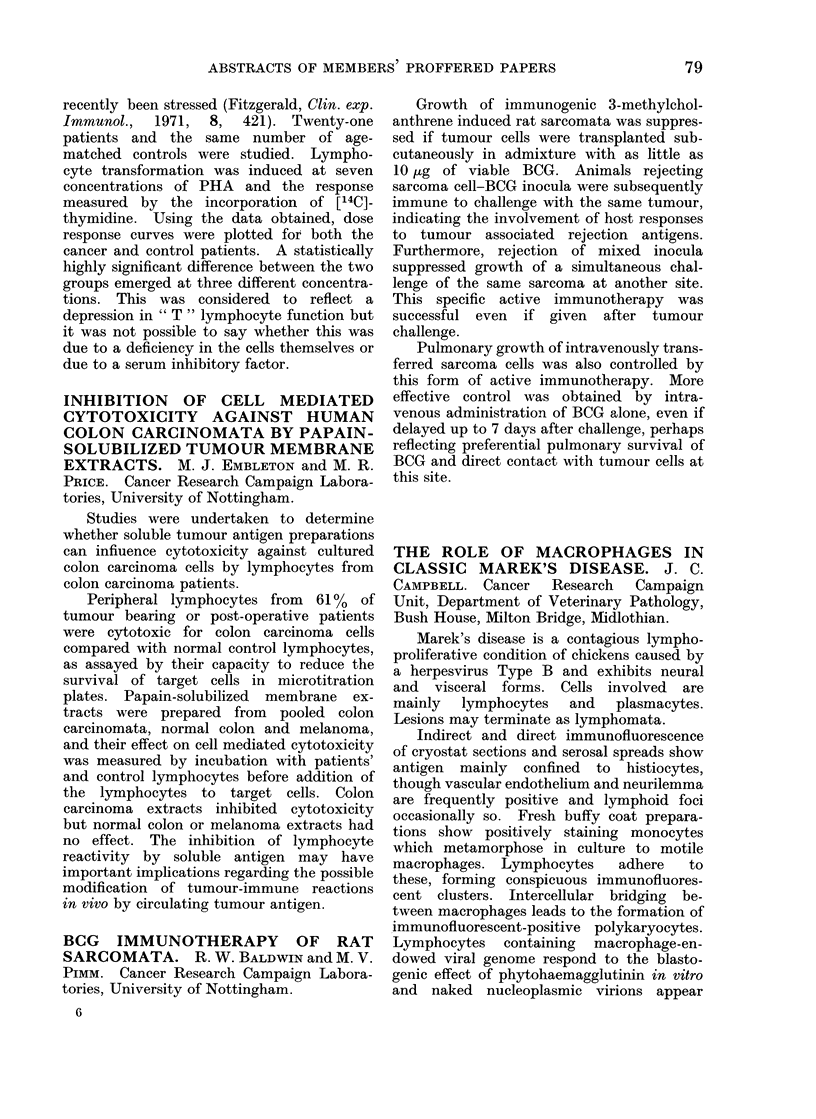# Inhibition of cell mediated cytotoxicity against human colon carcinomata by papain-solubilized tumour membrane extracts.

**DOI:** 10.1038/bjc.1973.83

**Published:** 1973-07

**Authors:** M. J. Embleton, M. R. Price


**INHIBITION OF CELL MEDIATED CYTOTOXICITY AGAINST HUMAN COLON CARCINOMATA BY PAPAIN-SOLUBILIZED TUMOUR MEMBRANE EXTRACTS.** M. J. Embleton and M. R. Price. Cancer Research Campaign Laboratories, University of Nottingham.

Studies were undertaken to determine whether soluble tumour antigen preparations can influence cytotoxicity against cultured colon carcinoma cells by lymphocytes from colon carcinoma patients.

Peripheral lymphocytes from 61% of tumour bearing or post-operative patients were cytotoxic for colon carcinoma cells compared with normal control lymphocytes, as assayed by their capacity to reduce the survival of target cells in microtitration plates. Papain-solubilized membrane extracts were prepared from pooled colon carcinomata, normal colon and melanoma, and their effect on cell mediated cytotoxicity was measured by incubation with patients' and control lymphocytes before addition of the lymphocytes to target cells. Colon carcinoma extracts inhibited cytotoxicity but normal colon or melanoma extracts had no effect. The inhibition of lymphocyte reactivity by soluble antigen may have important implications regarding the possible modification of tumour-immune reactions *in vivo* by circulating tumour antigen.